# HIV-1 envelope sequence-based diversity measures for identifying recent infections

**DOI:** 10.1371/journal.pone.0189999

**Published:** 2017-12-28

**Authors:** Alexis Kafando, Eric Fournier, Bouchra Serhir, Christine Martineau, Florence Doualla-Bell, Mohamed Ndongo Sangaré, Mohamed Sylla, Annie Chamberland, Mohamed El-Far, Hugues Charest, Cécile L. Tremblay

**Affiliations:** 1 Département de microbiologie, infectiologie et immunologie, Faculté de médecine, Université de Montréal, Montréal, Québec, Canada; 2 Laboratoire de santé publique du Québec, Institut national de santé publique du Québec, Sainte-Anne-de-Bellevue, Québec, Canada; 3 Department of medicine, division of experimental medicine, McGill University, Montreal, Québec, Canada; 4 Département de médecine sociale et préventive, École de santé publique, université de Montréal, Montréal, Québec, Canada; 5 Centre de recherche du centre hospitalier de l’Université de Montréal, Montréal, Québec, Canada; "INSERM", FRANCE

## Abstract

Identifying recent HIV-1 infections is crucial for monitoring HIV-1 incidence and optimizing public health prevention efforts. To identify recent HIV-1 infections, we evaluated and compared the performance of 4 sequence-based diversity measures including percent diversity, percent complexity, Shannon entropy and number of haplotypes targeting 13 genetic segments within the *env* gene of HIV-1. A total of 597 diagnostic samples obtained in 2013 and 2015 from recently and chronically HIV-1 infected individuals were selected. From the selected samples, 249 (134 from recent *versus* 115 from chronic infections) *env* coding regions, including V1-C5 of gp120 and the gp41 ectodomain of HIV-1, were successfully amplified and sequenced by next generation sequencing (NGS) using the Illumina MiSeq platform. The ability of the four sequence-based diversity measures to correctly identify recent HIV infections was evaluated using the frequency distribution curves, median and interquartile range and area under the curve (AUC) of the receiver operating characteristic (ROC). Comparing the median and interquartile range and evaluating the frequency distribution curves associated with the 4 sequence-based diversity measures, we observed that the percent diversity, number of haplotypes and Shannon entropy demonstrated significant potential to discriminate recent from chronic infections (p<0.0001). Using the AUC of ROC analysis, only the Shannon entropy measure within three HIV-1 *env* segments could accurately identify recent infections at a satisfactory level. The *env* segments were gp120 C2_1 (AUC = 0.806), gp120 C2_3 (AUC = 0.805) and gp120 V3 (AUC = 0.812). Our results clearly indicate that the Shannon entropy measure represents a useful tool for predicting HIV-1 infection recency.

## Introduction

At the end of 2014, the Public Health Agency of Canada (PHAC) estimated that approximately 75,500 people were living with HIV/AIDS in Canada, of whom 21% were unaware of their status [1]. Some efforts are made nowadays to increase diagnosis and linkage to care to newly diagnosed persons. But it is important to differentiate individuals who were recently infected from those with chronic infection as it may have a different impact on the epidemic and its control. It is estimated that the probability of HIV transmission is 26 times higher during the first 3 months of infection [2, 3] due to the high viral load in newly infected individuals [2–7]. Hence, identifying recently infected individuals is not only a key measure for better estimating HIV-1 incidence within the general population [4, 6, 8–10], but is also a valuable tool for monitoring HIV-1 epidemics and optimizing prevention efforts [3–6] to reduce HIV-1 forward transmission [2, 11–13].

Several strategies have been developed to identify recent infections, each exhibiting a varying efficacy [4, 14–18]. The traditional epidemiological method consists of prospectively following-up HIV infection rates within cohorts of HIV-1 negative individuals presenting with a high risk of HIV infection [4, 18]. This approach requires complicated logistics, is expensive and leads to outcome results that are not representative of the situation prevailing in the general population [18–22]. Serology-based methods consist of evaluating biomarkers, such as the presence of antigens and specific antibodies, and their modulation in time [23–35].

A multi-assay based serological algorithm based on two commercially available avidity assays [24] was recently developed in our laboratory. It has been shown to provide good discriminatory power to identify individuals infected within 136 days mean duration of recent infection (MDRI), with an estimated false recency rate of 3.3% [24]. This algorithm was used in the present study to classify clinical specimens as recently infected individuals (MDRI < 136 days) or chronically infected individuals (>136 days) [24]. Finally, a variety of molecular-based assays monitoring the HIV-1 viral genetic diversity throughout disease progression have been described, including: 1) The High Resolution Melting Assay (HRM), which evaluates the melting temperatures of HIV amplicons to estimate the number of HIV-1 quasi-species present in a given individual specimen [36–38]; 2) the number of ambiguous nucleotides (mixed bases) [17, 39], for which DNA sequences are usually provided by first generation sequencing; 3) the Hamming Distance (HD), which measures points of variation between two sequences of equal length [40, 41] using first generation sequencing; and 4) sequence-based diversity measurements as assessed by next generation sequencing (NGS) [42, 43], which is able to detect minor variants/mutations at low rates [44]. NGS is a powerful tool for evaluating HIV-1 sequence-based diversity [45] and was previously shown to be more accurate at detecting recent infections than any other molecular-based method [46]. In this study, HIV-1 *env* gene sequences, rather than those from *pol* or *gag*, were analyzed, as they are known to evolve more rapidly than other HIV-1 gene sequences[47–49]. The *env* diversity has already been shown to correlate with the HIV-1 Fiebig stages [50].

The HIV-1 envelope is a complex trimeric glycoprotein located on the viral surface and composed of the gp120 and gp41 subunits [47–49]. The gp120 subunit is subdivided into five conserved sub-domains (C1–C5) and five hyper-variable glycosylated loops (V1-V5) [51–54]. The gp41 subunit consists of an ectodomain (ECD), transmembrane domain (TM), and long cytoplasmic domain (CP) [55]. Each HIV-1 *env* subdomain or region plays a specific role in pathogenesis [52, 54, 56] and is differentially impacted by selective pressure. The present study evaluated the capacity to predict HIV-infection recency using four sequence-based diversity measures including the percent diversity, percent complexity, Shannon entropy and number of haplotypes, screening 13 HIV-1 *env* segments throughout the gp120 V1-C5 and the gp41 ectodomains.

## Materials and methods

### Patients and specimens

#### HIV positive samples

In the province of Québec (Canada), all serum samples that are repeatedly reactive using a screening HIV-1,2 enzyme immunoassay (EIA) are submitted to the provincial reference microbiology laboratory “(Laboratoire de Santé publique du Québec (LSPQ)” for confirmation mainly via a HIV-1 Western blot (WB) and/or HIV-1 p24 EIA. Western blot positive samples are submitted to a multi-assay algorithm (MAA) that combines a Centers for Disease Control and Prevention (CDC) modified Bio-Rad-Avidity assay followed by the Sedia-LAg-Avidity assay [24]. This MAA previously demonstrated good performance for identifying recent HIV-1 infections, showing a false recent rate (FRR) of 3.3% for a mean duration of recent infection (MDRI) of 136 days [24]. After the WB and EIA assays, residual sample volumes are stored at -20°C. For this study, recent infection samples were defined as follows: WB Negative or indeterminate, positive for HIV-1 p24 or positive according to WB but determined by MAA to be recent (within 136 days of infection). Established infection (chronic) samples were those that were positive according to WB and determined by MAA to be longstanding (> 136 days of infection).

A total of 164 recent (including 26 p24 antigen positives) and 154 chronic infection samples collected in 2013 as well as 117 recent (including 28 p24 antigen positives) and 162 chronic samples collected in 2015 were evaluated.

### Amplification and sequencing

Total nucleic acids were extracted from 100 μl of serum using an automated BioRobot MDx extraction platform using the QIAamp^®^Virus BioRobot^®^ MDx Kit (QIAGEN, Valencia, CA, USA). HIV-1 RNA was amplified using the Superscript III One-Step RT-PCR system with Platinium^®^ Taq DNA polymerase (Invitrogen, Thermo Fisher Scientific, Carlsbad, CA, USA) and the primers *env-up forward* (5’-GTTTCTTTTAGGCATCTCCTATGGCAGGAAGAAG-3’, HXB2 positions 5957–5983) and *env-lo**reverse* (5’-GTTTCTTCCAGTCCCCCCTTTTCTTTTAAAAAG-3’, HXB2 positions 9063–9088)[57]. The amplification conditions were as follows: 53°C for 30 minutes (for reverse transcription) and 94°C for 2 minutes for Taq DNA polymerase activation, followed by 40 cycles at 94°C for 15 s, 55°C for 30 s, and 68°C for 4 min. Nested amplification was performed using the Expand^™^ High Fidelity PCR System kit (Roche Diagnostics, Indianapolis, USA) as described by the manufacturer. The primers E60F forward (5’- TAATCAGTTTATGGGATCAAAGC -3’, HXB2 nucleotides positions 6547–6569) [58] and E55R reverse (5’-GCCCCAGACTGTGAGTTGCAACAGATG-3’, HXB2 nucleotides positions 7940–7914) [59] were used. The amplification conditions were: 94°C for 2 min, followed by 45 cycles at 94°C for 15 s, 55°C for 30 s, and 68°C for 2 min. PCR products were visualized by agarose gel electrophoresis and purified using the QIAquick 96 PCR Purification Kit from QIAGEN (QIAGEN, Valencia, CA, USA).

The nested RT-PCR generated ≈ 1400 bp of the *env* gene. For next generation sequencing (NGS), one nanogram (1 ng) of DNA quantified using the Quant-iT^™^ PicoGreen^®^ dsDNA Assay kit (Life technologies, Oregon, USA) was used for library preparation using the Nextera XT DNA library preparation kit from Illumina (Illumina, San Diego, CA) following the manufacturer’s protocol. DNA sequencing was performed on a MiSeq instrument (Illumina, San Diego, CA, USA) using MiSeq^®^ Reagent Kits v3 (600 cycles) following the manufacturer’s protocols.

### Sequence data processing and genetic diversity calculation

The quality of the NGS runs was evaluated using the Illumina Sequencing Analysis Viewer v1.10.2 Software and the FastQC application (http://www.bioinformatics.babraham.ac.uk/projects/fastqc/). Sequencing depth and coverage were available under Coverage.txt and ComputeGP120Coverage.sh in OneDrive HIV_A_kafando project following these links respectively: https://onedrive.live.com/?authkey=%21AB4CmrTlu182Xw8&cid=709AAE8E69A7368F&id=709AAE8E69A7368F%21361&parId=709AAE8E69A7368F%21351&o=OneUp. A species with a coverage less than 100x were excluded in final statistical analyses.

**Fig 1:**
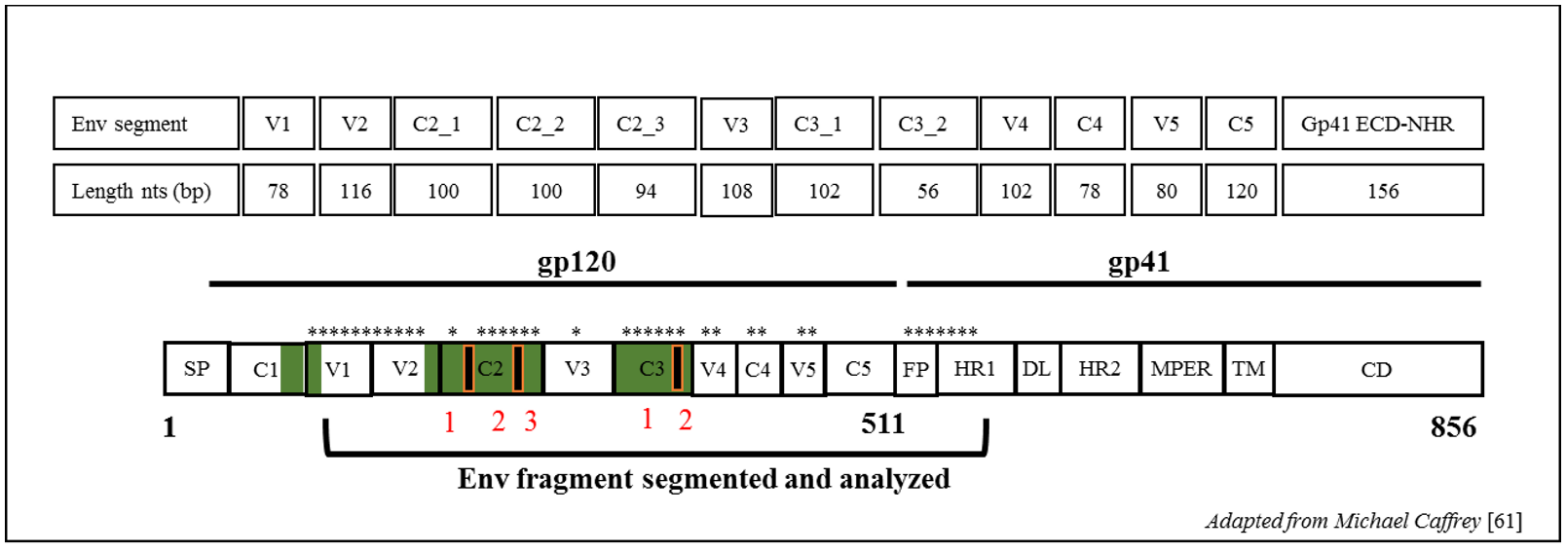
Schematic figure showing all *env* segments used for diversity estimates. Segments length corresponds to that of strain HXB2 of HIV-1 nucleotides positions. Segments used are denoted by asterisks. Env domain abbreviations: SP, signal peptide; C1–C5, conserved domains 1 to 5; V1–V5, variable domains 1 to 5; FP, fusion peptide; HR1, heptad repeat 1 (NHR); DL, disulfide loop; HR2, heptad repeat 2 (CHR); MPER, membrane proximal ectodomain region; TM, transmembrane domain; CD, cytoplasmic domain. Image were friendly adapted from Michael Caffrey[61]; Trends in Microbiology, Volume 19, Issue 4, Pages 191–197 (April 2011) 10.1016/j.tim.2011.02.001.

Sequences were *de novo* assembled using Iterative Virus Assembler (IVA) [60] to generate a consensus. The HIV-1 *env* subdomains gp120-V1 to C5 and a part of the gp41 ectodomain (first 158pb) were analyzed separately. The gp120-C2 and C3 subdomains were subdivided into 3 and 2 segments for subsequent analyses to compare DNA sequences of sizes like the other regions as showed in Fig 1.

To map subdomains, consensus sequences were aligned with the HXB2 *env* reference sequence (Genbank accession number K03455.1-HIVHXB2CG *env* nucleotides positions 6225–8795) using Clustal W in MEGA7.0 (www.megasoftware.net) [62].

The *env* subdomain length delimitations followed the HXB2 complete genome numbering were as follows: gp120 V1 (6615–6692 ≈78pb), V2 (6696–6812≈116pb), C2_segment 1 (6813–6913≈100pb), C2_segment 2 (6914–7014≈100pb), C2_segment 3 (7015–7109≈94pb), V3 (7110–7217≈108pb), C3_segment 1 (7218–7320≈102pb), C3_segment 2 (7321–7376≈56pb), V4 (7377–7478≈102pb), C4 (7479–7556≈78pb), V5 (7557–7637≈80pb), C5 (7638–7757≈120pb) and gp41-ectodomain (7758–7915≈158pb).

Intra-patient genetic diversity was evaluated for each subdomain/segment using an in-house coded Python pipeline. SMALT (http://www.sanger.ac.uk/science/tools/smalt-0) was used to map the reads against their respective consensus sequence, and SAM tools (Sequence Alignment/Map)[63] were used for analysis of the mapping file generated by SMALT. Bioconductor packages (https://bioconductor.org/) [64] were used for the genetic diversity calculation. More details about the specific packages and the python codes used for diversity estimates are available and DOIs to access are below: https://github.com/EricFournier3/HIVvariant and https://1drv.ms/f/s!Ao82p2mOrppwgl8eApq05btfNl8P

The four sequence-based diversity measures were calculated as previously described [36, 42] as show in S1 Table. Briefly, the percent diversity was evaluated as the average pairwise genetic distance between two sequences [42], the percent complexity was expressed as the number of distinct variants divided by the total number of reads x 100 [42], and the Shannon entropy index (S) was calculated using a formula that accounts for both the number of distinct reads and their proportional representation in the dataset [42, 43]. The number of haplotypes strictly included the number of distinct quasi-species or variants present in at least 1% or more in the viral population [42]. The frequency distribution curves (ggplot2) of the percent diversity, percent complexity, Shannon entropy and number of haplotypes for recent *versus* chronic sequences were generated using R [65].

### Determination of HIV subtypes

We used two HIV subtyping tools to determine a consensus HIV subtype. The Rega HIV Subtyping Tool V3 [66] (http://regatools.med.kuleuven.be/typing/v3/hiv/typingtool) and, Confirmation with the NCBI HIV Subtyping tool [67] (https://www.ncbi.nlm.nih.gov/projects/genotyping/formpage.cgi).

### Determination of the sequence-based diversity measure performance

The performance of individual or combined sequence-based diversity measures for discriminating recent from chronic infections was evaluated using the area under the curve (AUC) of the receiver operating characteristics (ROC) [68]. The best value for the AUC is 1.0, which represents 100% sensitivity and 100% specificity at distinguishing recent from chronic infections. Interpretations of the AUC values for the sequence-based diversity of recent *versus* chronic HIV-1 infected individuals in our study followed the grading guidelines that were previously described by D.G Kleinbaum and M. Klein (2012) [68]. Briefly, scores from 0.90–1.0 were excellent discrimination (scored A), scores from 0.80–0.90 good discrimination (scored B), scores from 0.70–0.80 fair discrimination (scored C), scores from 0.60–0.70 poor discrimination (scored D) and scores from 0.50–0.60 failed discrimination (scored F). AUC of ROC analysis was also used to identify the optimal cut-off values that would distinguish recent from chronic infections with high accuracy (high sensitivity and high specificity) as previously described [69]. The following online link will help understanding calculation methods and identification of the optimal or best cut-off values: https://ncss-wpengine.netdna-ssl.com/wp-content/themes/ncss/pdf/Procedures/NCSS/One_ROC_Curve_and_Cutoff_Analysis.pdf. The best cut-off values of measures must have the highest accuracy, which corresponds to its capacity to correctly classify the highest true positive rate (TPR) or sensitivity and highest true negative rate (TNR) or specificity. Here, the TPR represents the recent HIV-1 infected individuals who were correctly classified and the TNR represents the chronic HIV-1 infected individuals who were also correctly classified by the same test.

### Statistical analyses

Summary statistics (mean, median and interquartile range) were used to estimate the intra and inter-patient envelope genetic diversity.

The student t-test was used to compare the diversity measures between sequences from recent and chronic infections. Analyses were performed using Epi Info^™^ 7 (www.cdc.gov/epiinfo) and IBM SPSS Statistics software. P-values below 0.05 were considered statistically significant.

### Ethics statement

A retrospective patient’s samples were used in this study and were obtained from the LSPQ serobank collection. They were collected for routine diagnostic purposes in 2013 and 2015. All sample were anonymized before we accessed them for the study. No nominals information’s of patient were used for analysis and data management. Written informed consent was obtained from individuals in the primary HIV infection (PHI) cohort of Quebec included in study. Ethical clearance was obtained from the “Le Comité d′éthique de la recherche (CÉR) du Centre hospitalier de l′Université de Montréal (CHUM), Montreal, Canada.

### Nucleotide sequence accession number

The Miseq d’Illumina sequencing data obtained in this study (n = 249) were deposited and available in the GenBank Sequence Database (NCBI) under GenBank accession **KY946451 to KY946713** as reported in S1 Dataset.

## Results

**Fig 2:**
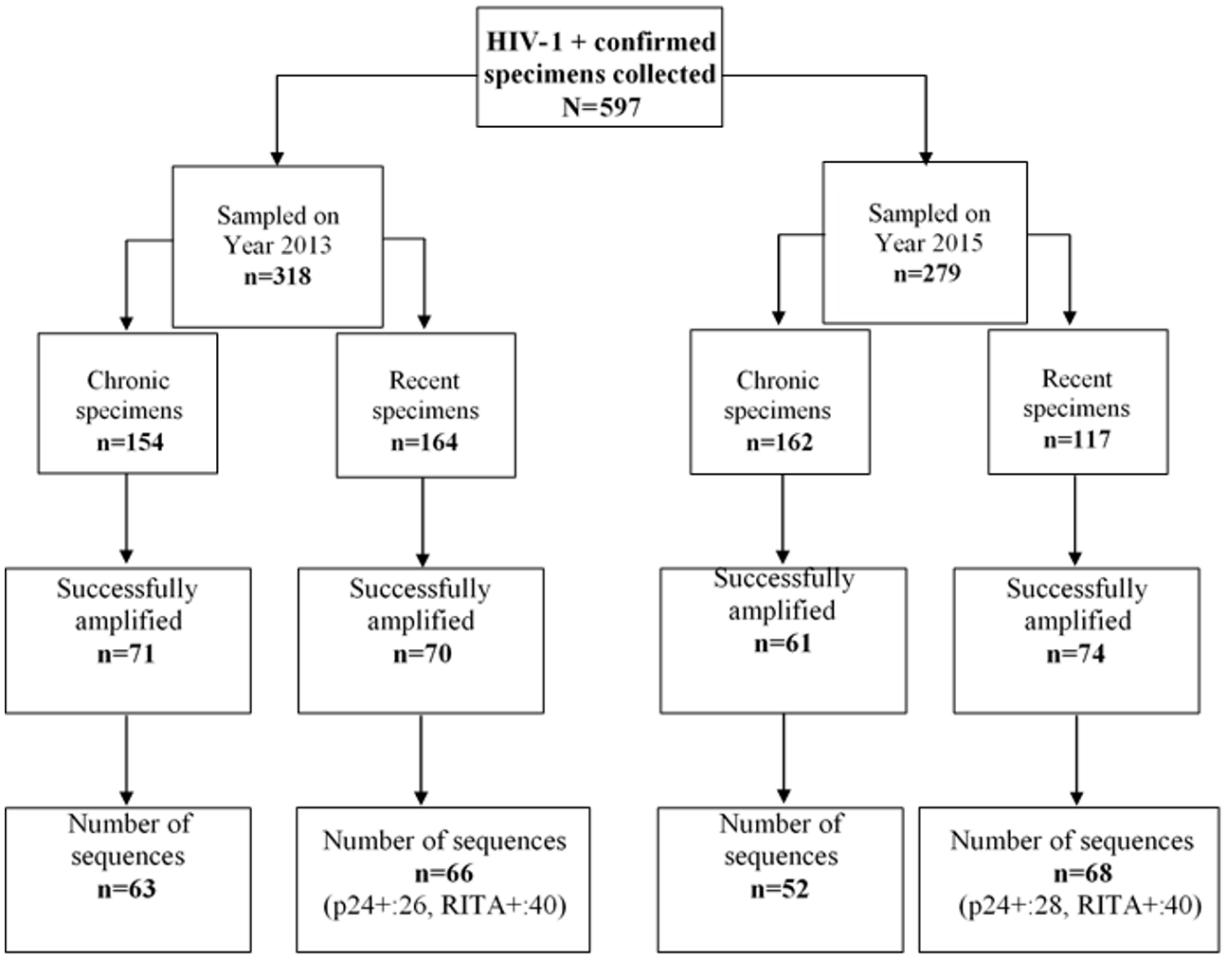
Number of sequences (one per patient) used in this study. N = 249 derived from 134 recently versus 115 chronically HIV-1 infected individual’s sequences data were included in the study.

A total of 597 specimens from individual newly diagnosed HIV-1+ and sampled in years 2013 and 2015 were collected in this study. All, except for p24 positive samples, were subjected to an avidity Multi-assay algorithm (MAA) to assess infection recency by serological tests. From those, 281 were categorized as recent infections and 316 as chronic. These specimens were not successfully passed the PCR amplification and sequencing process. The success rate of the nested RT-PCR step was 46% (276/597), and 97% of the latter were successfully sequenced (n = 263) as presented in Fig 2. At the sequences data management and processing, the very shorts ones or containing gaps estimates to 5% (14/263) after alignment with HXB2 *env* reference sequence were excluded. Finally, n = 249 sequences that corresponds to one per patient were included in this current study (Fig 2 and S1 Dataset).

For HIV-1 subtyping, seventy-seven per cent (77%) of the sequenced samples (n = 192/249) were clade B subtypes and 30% for non-B as presented in Table 1 and reported in S1 Dataset.

**Table 1:** HIV-1 subtype distribution of the sequences analyzed in this study.

HIV-1 Subtype	Number of sequences for recently HIV-1 infected individuals		Number of sequences for chronically HIV-1 infected individuals		Total of number of sequences	
	No	%	No	%	No	%
**A1**	11	8,21%	12	10,43%	23	9,24%
**B**	**106**	**79,10%**	**86**	**74,78%**	**192**	**77,11%**
**C**	3	2,24%	6	5,22%	9	3,61%
**CRF 01_AE**	7	5,22%	0	0,00%	7	2,81%
**CRF 02_AG**	1	0,75%	3	2,61%	4	1,61%
**CRF11**	0	0,00%	1	0,87%	1	0,40%
**D**	5	3,73%	2	1,74%	7	2,81%
**F1**	1	0,75%	3	2,61%	4	1,61%
**G**	0	0,00%	2	1,74%	2	0,80%
**TOTAL**	134	100%	115	100%	249	100%

**Fig 3:**
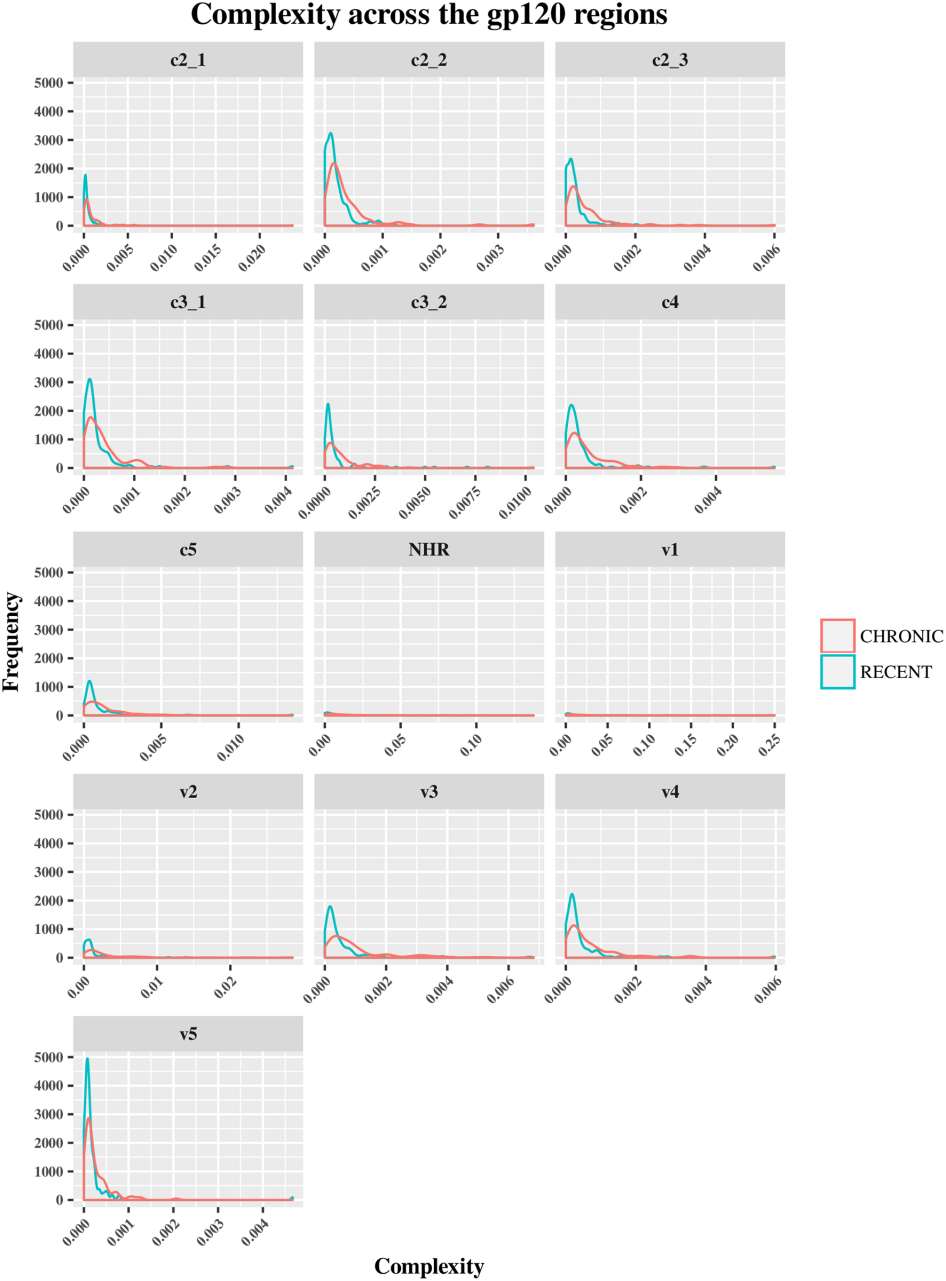
Frequency polygons (ggplot2) of percent complexity of *env* sequences of recent HIV-1 infected individuals compare to chronically infected ones by *env* segments. The Y axis represents the density of observations (frequency) and the X axis the percent complexity distribution as sequence-based diversity measure. The blue color represents plot and distribution for recent HIV-1 infected population and the red color plot and distribution for chronic infected ones.

**Fig 4:**
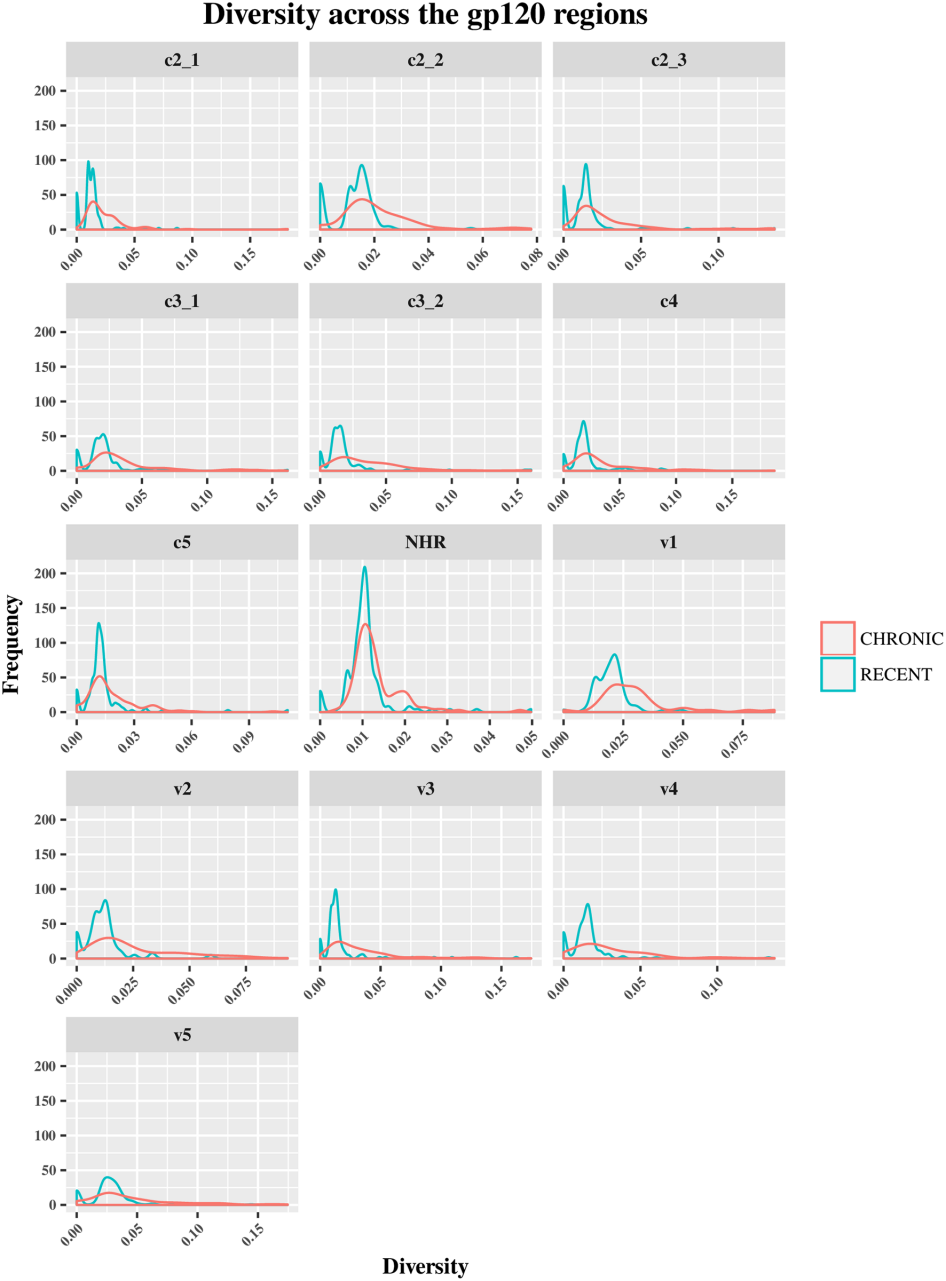
Frequency polygons (ggplot2) of percent diversity of *env* sequences of recent HIV-1 infected individuals compare to chronically infected ones by *env* segments. The Y axis represents the density of observations (frequency) and the X axis the percent diversity distribution as sequence-based diversity measure. The blue color represents plot and distribution for recent HIV-1 infected population and the red color plot and distribution for chronic infected ones.

**Fig 5:**
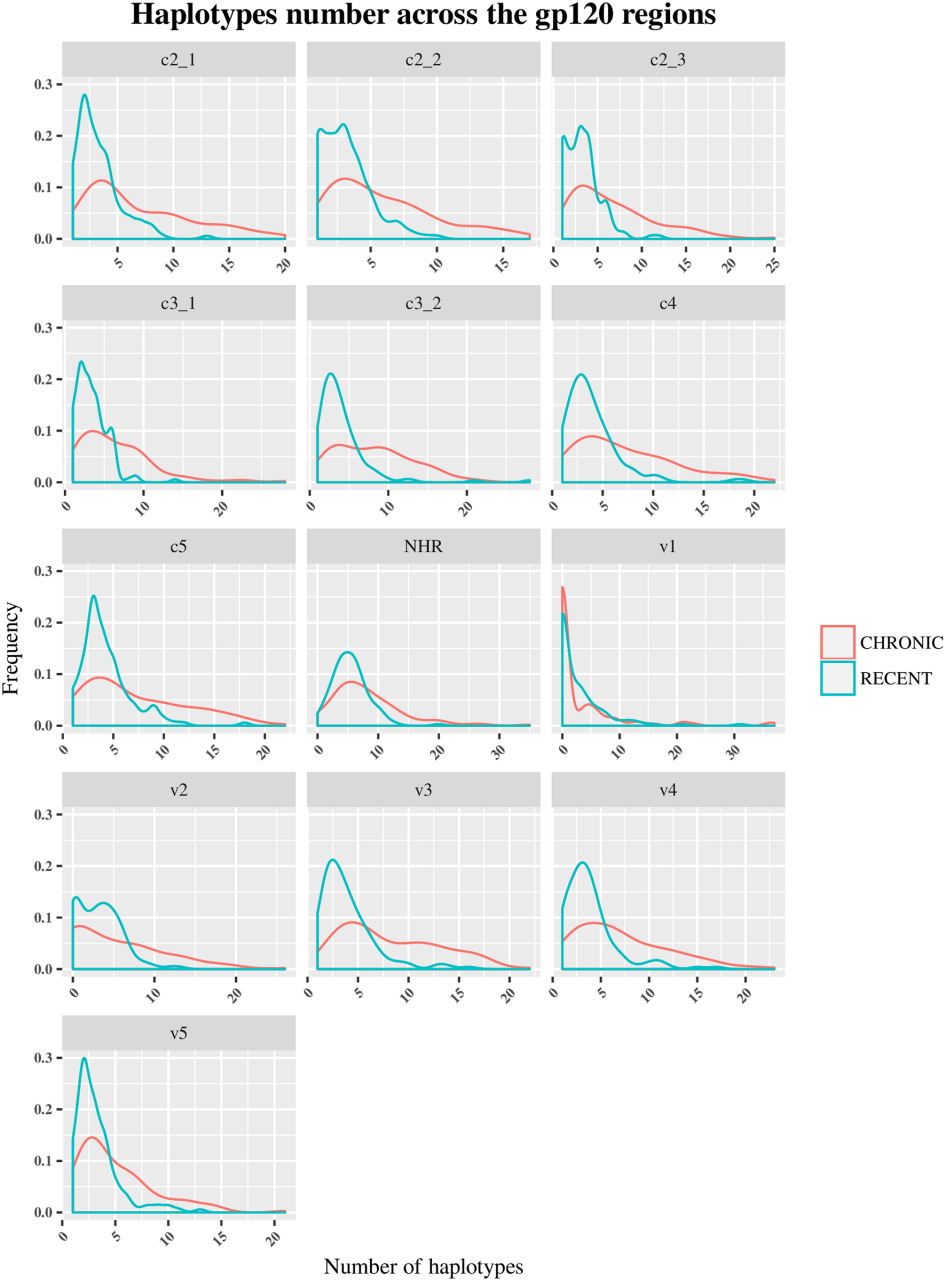
Frequency polygons (ggplot2) of number of haplotypes of *env* sequences of recent HIV-1 infected individuals compare to chronically infected ones by *env* segments. The Y axis represents the density of observations (frequency) and the X axis the number of Haplotypes distribution as sequence-based diversity measure. The blue color represents plot and distribution for recent HIV-1 infected population and the red color plot and distribution for chronic infected ones.

**Fig 6:**
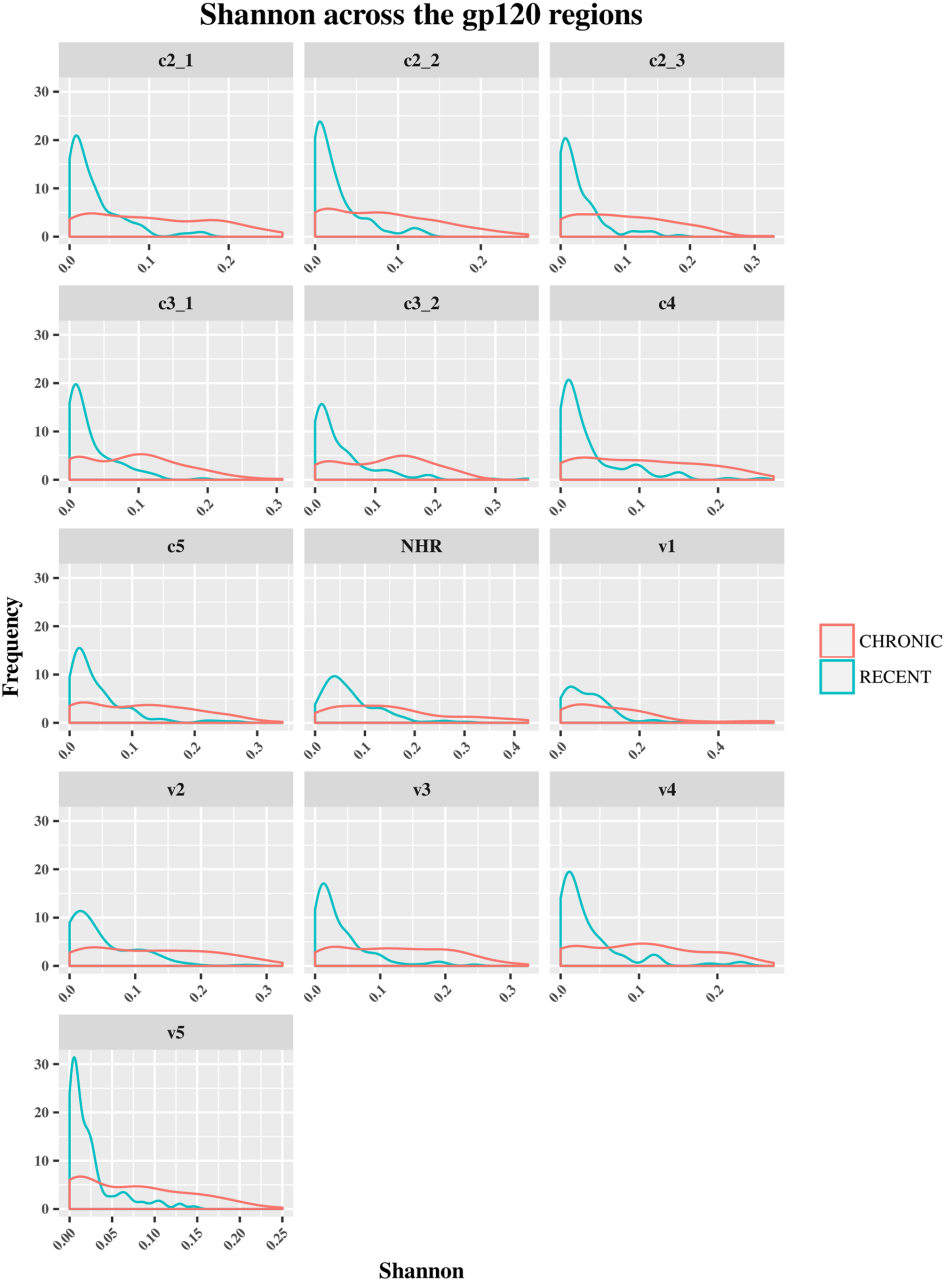
Frequency polygons (ggplot2) of Shannon entropy index of *env* sequences of recent HIV-1 infected individuals compare to chronically infected ones by *env* segments. The Y axis represents the density of observations (frequency) and the X axis the Shannon entropy index distribution as sequence-based diversity measure. The blue color represents plot and distribution for recent HIV-1 infected population and the red color plot and distribution for chronic infected ones.

The HIV-1 *env* diversity in specimens from recent and chronic infections was examined using four distinct sequence-based diversity measures including percent diversity, percent complexity, Shannon entropy and number of haplotypes. To study the profile of diversity values that are associated with recent *versus* chronic specimens, frequency distribution curves (ggplot2 of R) were generated for each of the 4 sequence-based diversity measures and across all the HIV-1 *env* segments selected (Figs 3–6).

These frequencies of diversity distribution curves for each measure comparing recent to chronic HIV-1 infected individuals by *env* segment are also available online following this link: https://1drv.ms/f/s!Ao82p2mOrppwgl8eApq05btfNl8P.

As shown in Fig 3, the percent complexity frequency distribution curves from recent *versus* chronic infections were overlapped at the same low complexity level. These results showed that this diversity measure did not allow for a clear discrimination between the two HIV-1 infected populations. The medians and means analyses of diversity seemed to confirm these observations for all the HIV-1 e*nv* segments analyzed (S2, S3 and S4 Tables). But, A statistical analysis (student t-test) confirm this view for only *env* gp120 C3_2, V1 and V5 segments (P>0.05), the others *env* segments were statistically significant(P<0.05) as summarized in S4 Table.

Conversely, the percent diversity (Fig 4), number of haplotypes (Fig 5) and Shannon entropy (Fig 6) distribution curves of recently infected individual’s sequences peaked at lower diversity values compared to the curves associated with chronic infections, which were more widely distributed and shifted towards higher diversity values for all the *env* segments analyzed. These observations were indicative of the good discriminatory power of these 3 diversity measures for all the *env* segments tested. The summary statistics (mean, medians and IQR observations seemed to confirm the differences between recent and chronic HIV-1 infected populations as showed in S2, S3 and S4 Tables.

Also, the statistical analyses using student t-test confirms and demonstrates a significant difference between recent and chronic sequences diversity (P<0.05) for these 3 measures for any *env* segments analyzed as showed in S4 Table.

**Fig 7:**
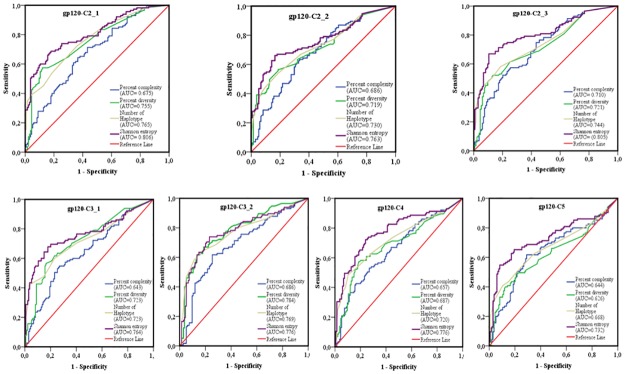
ROC curves comparing the performance of the 4 sequence-based diversity measures for discriminating recent from chronic HIV-1 infection. Four selected HIV-1 gp120 conserved subdomains (C2, C3, C4 and C5) subdivided on seven segments were analyzed, 3 segments on the gp120-C2 region (C2_1; C2_2 and C2_3), 2 segments on the gp120-C3 region (C3_1 and C3_2), 1 segment on gp120-C4 and 1 segment on gp120-C5. The Y axis represents the proportion of sequences from true recent HIV-1 infected individuals (sensitivity), and the X axis the proportion of recent HIV-1 infected individuals who were incorrectly classified (1-specificity). ROC = receiver operating characteristics. AUC (area under the curve) values between 0.8 and 1 were considered performance measures.

**Fig 8:**
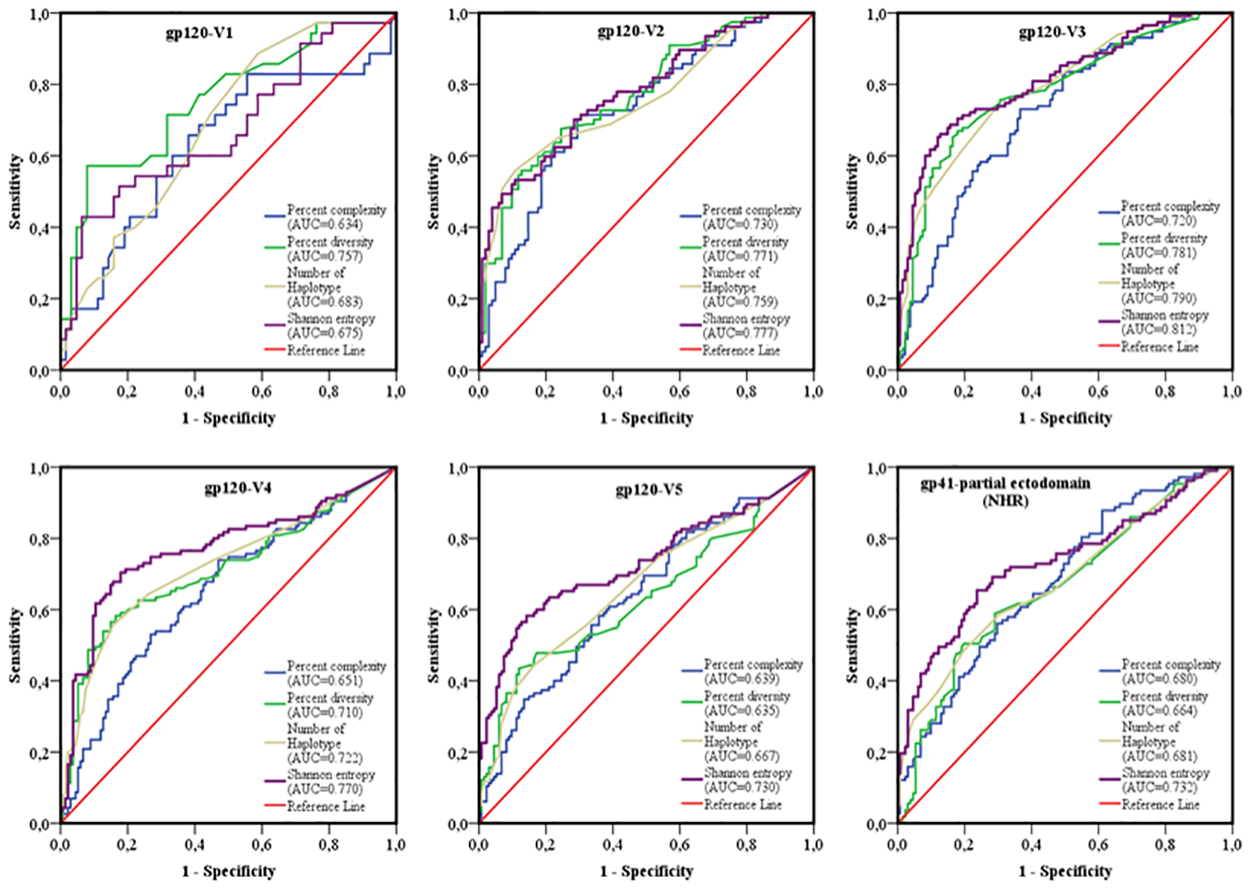
ROC curves comparing the performance of the 4 sequence-based diversity measures for discriminating recent from chronic HIV-1 infection. Five HIV-1 gp120 variable loops and one part of gp41 ectodomain (NHR). Five segments represented each of the HIV-1 gp120 variable loop as well as 1 segment of the gp41- NHR ectodomain were analyzed: gp120-V1 loop, gp120-V2 loop, gp120-V3 loop, gp120-V4 loop, gp120-V5 loop and part of the gp41-NHR ectodomain. The Y axis represents the proportion of sequences from true recent HIV-1 infected individuals (sensitivity), and the X axis represents the proportion of recent HIV-1 infected individuals who were incorrectly classified (1-specificity). ROC = receiver operating characteristics. NHR = N-terminal heptad repeat. AUC values between 0.8 and 1 were considered performance measures.

**Fig 9:**
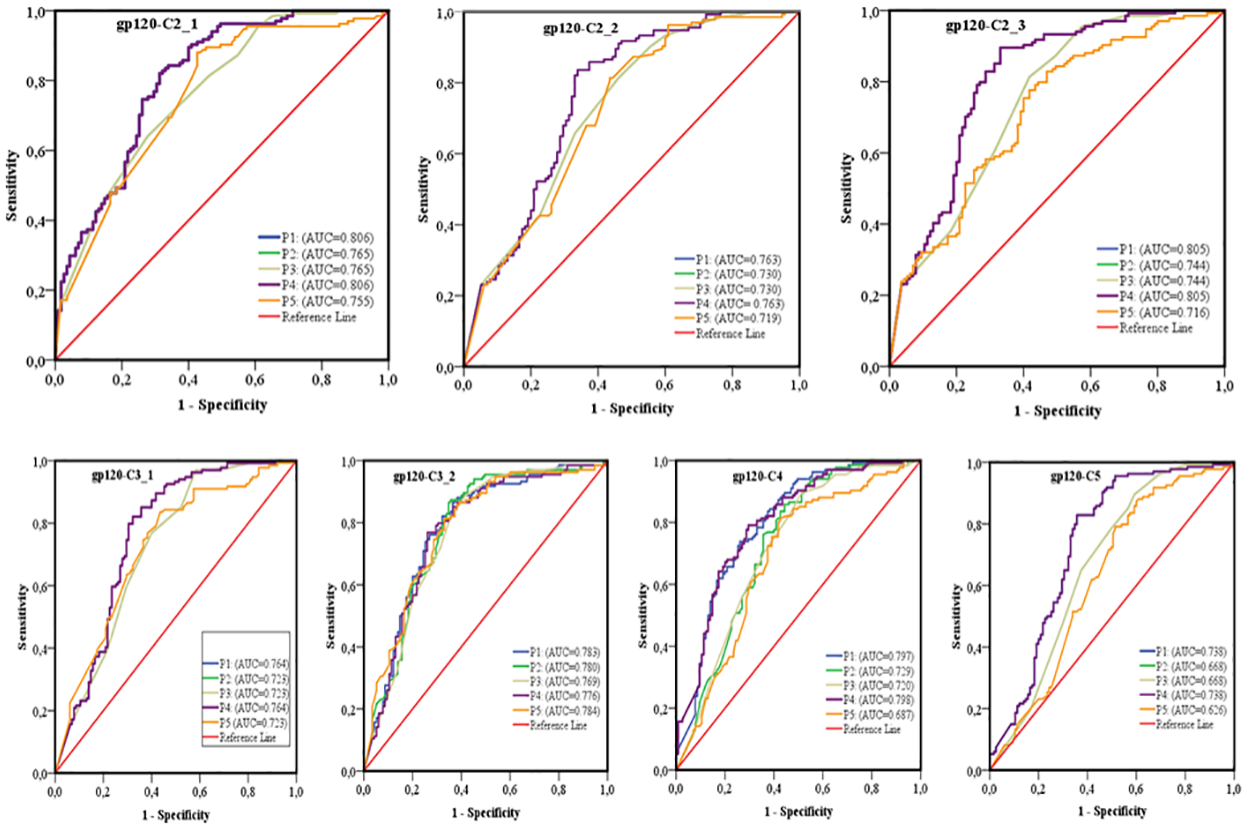
ROC curves comparing the predictive performance of different combinations of sequence-based diversity measures of HIV-1 gp120 conserved subdomains to identify HIV-1 infection recency. Five combinations of sequence-based diversity measures were analyzed. Shannon entropy + percent diversity + percent complexity: P1; percent diversity+ number of haplotypes+ percent complexity: P2; number of haplotypes+ percent complexity: P3; Shannon entropy+ percent complexity: P4 and percent diversity+ percent complexity: P5. Seven HIV-1 *env* segments were considered: gp120-C2_1; gp120-C2_2; gp120-C2_3; gp120-C3_1; gp120-C3_2; gp120-C4 and gp120-C5. ROC = receiver operating characteristics; AUC = area under the curve. AUC values between 0.8 and 1 were considered performance measures.

**Fig 10:**
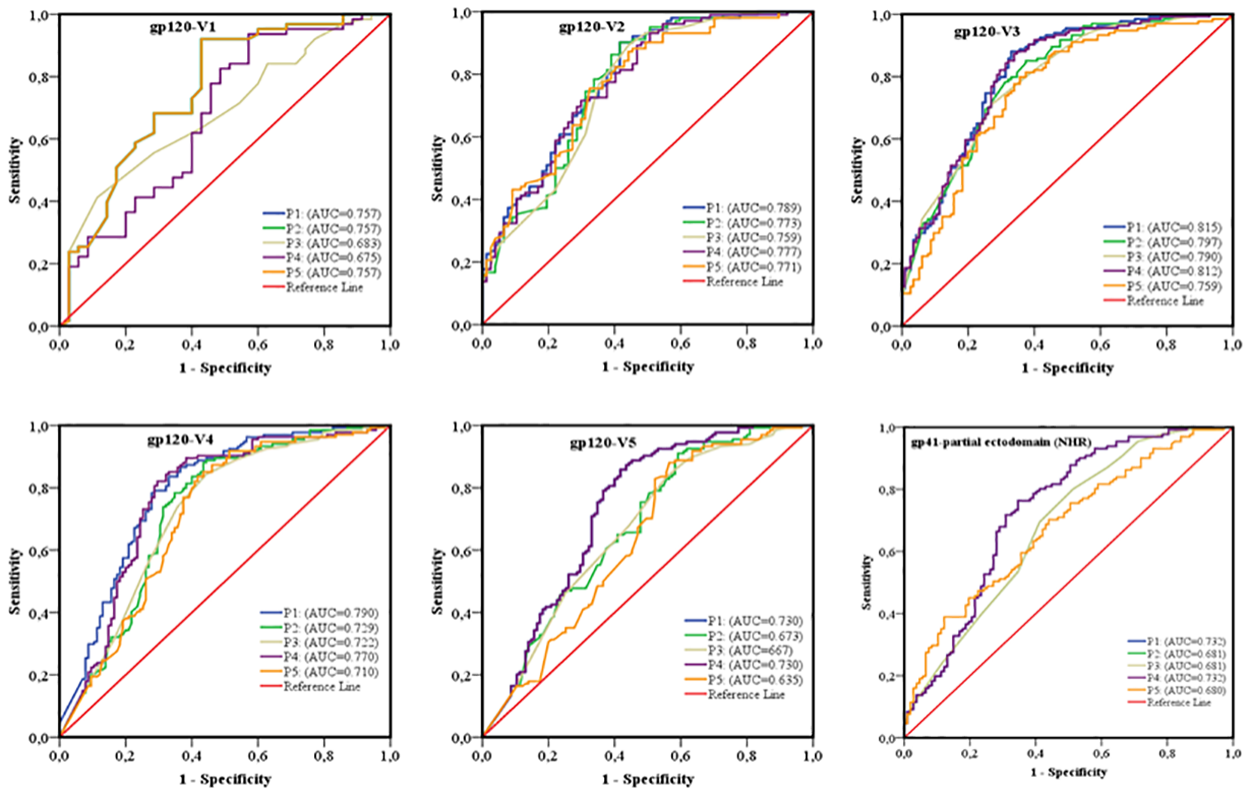
ROC curves comparing the predictive performance of different combinations of sequence-based diversity measures of five HIV-1 *env* gp120 variable loops and one part of the gp41-ectodomain (NHR) to identify HIV infection recency. Five combinations of sequence-based diversity measures were analyzed. Shannon entropy + percent diversity + percent complexity: P1; percent diversity+ number of haplotypes+ percent complexity: P2; number of haplotypes+ percent complexity: P3; Shannon entropy+ percent complexity: P4 and percent diversity+ percent complexity: P5. Six HIV-1 *env* segments were considered: gp120-V1 loop; gp120-V2 loop; gp120-V3 loop; gp120-V4 loop; gp120-V5 loop and, gp41-NHR (partial ectodomain). NHR = N-terminal heptad repeat. ROC = receiver operating characteristics; AUC = area under the curve. AUC values between 0.8 and 1 were considered performance measures.

**Fig 11:**
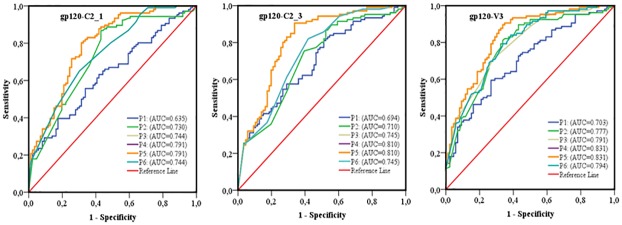
ROC curves comparing the predictive performance of different combinations of sequence-based diversity measures of HIV gp120-C2-1, gp120-C2_3 and gp120-V3 segments for identifying HIV-1 subtype B infection recency. Five combinations of sequence-based diversity measures were analyzed: P1, percent complexity; P2, percent diversity; P3, number of haplotypes; P4, Shannon entropy; P5, Shannon entropy+ percent diversity and P6, Number of haplotypes+ percent diversity. Three HIV-1 env segments were considered: gp120-C2_1, gp120- C2_3 and gp120-V3 -. ROC = receiver operating characteristics; AUC = area under the curve. AUC values between 0.8 and 1 were considered performance measures.

The area under the curve (AUC) of receiver operating characteristics (ROC) analysis was used to compare the performance of each sequence-based diversity measure in their ability to identify HIV-1 infection recency based on analysis of the 13 segments of HIV-1 *env* (Figs 7 and 8). Using the percent complexity measure, we observed that both the gp120-V2 and V3 loop segments showed fair discrimination (AUC = 0.7) as opposed to the other eleven env segments tested, which presented poor discrimination (AUC≤ 0.6). Using the percent diversity measure, fair discrimination was observed for 11 *env* segments (AUC = 0.7), while the 3 *env* segments, gp120- C4, C5 and V5, exhibited poor discrimination (AUC≤0.6). The number of haplotypes measures exhibited fair discrimination for 10 env segments (AUC = 0.7) and poor discrimination for 3 *env* segments, gp120 C5, V1 and V5 (AUC≤0.6). However, the Shannon entropy showed good discrimination power for 3 *env* segments, gp120 C2_1, C2_3 and V3 (AUC≥0.8); fair discriminatory power (AUC = 0.7) for 9 *env* segments, gp120 C2_1, C3_1, C3_2, C4, C5, V2, V4, V5 and gp41-ectodomain; and poor discriminatory power (AUC ≤ 0.6) for the *env* gp120 V1 segment. The Shannon entropy was the only single sequence based diversity measure for which a significantly good discriminatory power was observed. It is therefore identified as the best performing diversity measure for the HIV-1 *env* segments analyzed. More specifically, the gp120 C2_1, C2_3 and V3 HIV-1 *env* segments appeared to be the most predictive for identifying HIV-1 recency (Table 2 and Figs 7–11).

**Table 2:** Performance of Shannon entropy as a measure for identifying recent HIV-1 infections.

HIV-1 *env* segment	Diversity measure	Optimal Cut-off value	HIV-1 Subtype	AUC	AUC (95% CI)	TPR (Sn)	TNR (Sp)	N recent/chronic
GP120-C2_1	Shannon entropy	0.054	Multiple	0.806	[0.752–0.861]	82%	69%	134/115
GP120-C2_3	Shannon entropy	0.059	Multiple	0.805	[0.749–0.862]	90%	67%	134/115
GP120-V3	Shannon entropy	0.083	Multiple	0.812	[0.758–0.866]	87%	66%	134/115
GP120-C2_1	Shannon entropy	0.054	B	0.791	[0.726–0.856]	80%	69%	106/86
GP120-C2_3	Shannon entropy	0.06	B	0.810	[0.747–0.873]	91%	66%	106/86
GP120-V3	Shannon entropy	0.083	B	0.831	[0.773–0.889]	90%	66%	106/86
GP120-V3	Shannon entropy	0.083	Multiple:WB+/MAA+specimens as recent	0.801	[0.740–0.863]	89%	66%	80/115
GP120-V3	Shannon entropy	0.097	Multiple: p24+ specimens as recent	0.827	[0.763–0.891]	93%	60%	54/115
GP120-C2_1	Shannon entropy	0.06	Multiple: p24+ specimens as recent	0.850	[0.794–0.905]	93%	66%	54/115
GP12-C2_3	Shannon entropy	0.048	Multiple: p24+ specimens as recent	0.844	[0.786–0.902]	91%	71%	54/115

TPR (true positive rate) = sensitivity: recent HIV-1+ specimens correctly classified,

TNR (true negative rate) = specificity: chronic HIV-1+ specimens correctly classified. The optimal cutoff value indicates the proportion of patients correctly classified and represented the highest TPR (recency) + TNR (chronic) or (Sensitivity+ Specificity).

To increase the discriminatory power of our assays, we combined the 4 sequence-based diversity measures and used logistic regression analyses to identify the combination that performed best. As presented in Figs 9 and 10, only combinations including Shannon entropy, such as Shannon entropy + percent diversity + percent complexity, or Shannon entropy + percent diversity or Shannon entropy + percent complexity of gp120 V3 (AUC = 0.815), gp120 C2_1 (AUC = 0.806) and gp120 C2_3 (AUC = 0.805), presented a performance equivalent to that of Shannon entropy alone for the same respective *env* segments, gp120 V3 (AUC = 0.812), gp120 C2_1 (0.806) and gp120 C2_3 (0.805). The other sequence-based diversity measure combinations for any of the 13 *env* segments analyzed showed a fair to poor discriminatory power (Figs 9 and 10).

The *env* gp120 C2 and C3 sub regions as too long were previously segmented on 3 and 2 segments respectively to respect the objective of less than 100bp as sliding window for our analyses. As showed in Table 3, the combining multiple segments didn’t increase the discriminatory effect. For example, when combining gp120-C2 sub region including C2_1+C2_2+C2_3 segments the area under the curve (AUC) of ROC of Shannon entropy for example were: 0,790 CI95% [0,757–0,823] and less than this previous value for the 3 others measures. This Combination predicts moderate discriminatory effects compared to C2_1 and C2_3 alone which adequately predicted HIV recency with respectively AUC = 0.806 and AUC = 0.805. Combining *env* gp120 C3 (C3_1+C3_2 segments) or considering *env* gp120-V1C5 also does not perform well either (AUC<0.8).

**Table 3:** Performance of 4 sequence based diversity measures for non-B HIV-1 subtypes by *env* segments using AUC au ROC analysis.

Env segment	Diversity measure	AUC	95% CI	Optimal Cut-off	TPR (Recent)	TNR (Chronic)	N: recent/chronic)
**GP120 C2_1**	Percent complexity	0,805	[0,693–0,918]	0,0004	75%	72%	28/29
	Percent diversity	0,842	[0,743–0,941]	0,0231	100%	52%	28/29
	Shannon entropy index	0,844	[0,741–0,946]	0,0895	96%	66%	28/29
	Nb_haplotypes	0,826	[0,720–0,931]	8	96%	55%	28/29
**GP120 C3_2**	Percent complexity	0,759	[0,631–0,887]	0,0004	93%	55%	28/29
	Percent diversity	0,821	[0,705–0,938]	0,0216	93%	72%	28/29
	Shannon entropy index	0,795	[0,669–0,921]	0,0774	89%	72%	28/29
	Nb_haplotypes	0,849	[0,740–0,959]	7	93%	72%	28/29
**GP120-C3**	Percent complexity	0,718	[0,623–0,812]	0,0002	38%	62%	28/29
	Percent diversity	0,803	[0,720–0,886]	0,0222	88%	72%	28/29
	Shannon entropy index	0,767	[0,677–0,857]	0,0776	86%	64%	28/29
	Nb_haplotypes	0,812	[0,728–0,896]	7	95%	62%	28/29
**GP120 C4**	Percent complexity	0,698	|0,557–0,839]	0,0003	89%	55%	28/29
	Percent diversity	0,819	[0,702–0,936]	0,0235	96%	66%	28/29
	Shannon entropy index	0,752	[0,622–0,883]	0,1214	100%	48%	28/29
	Nb_haplotypes	0,756	[0,625–0,887]	7	96%	55%	28/29
**GP120 V2**	Percent complexity	0,762	[0,621–0,903]	0,0012	79%	67%	28/29
	Percent diversity	0,845	[0,732–0,959]	0,0174	92%	67%	28/29
	Shannon entropy index	0,766	[0,622–0,909]	0,1338	92%	62%	28/29
	Nb_haplotypes	0,761	[0,615–0,907]	8	92%	57%	28/29

TPR (true positive rate) = sensitivity: recent HIV-1+ specimens correctly classified. TNR (true negative rate) = specificity: chronic HIV-1+ specimens correctly classified. The optimal cutoff value indicates the highest accuracy (proportion of patients correctly classified) represented the highest TPR (recency) + TNR (chronic) or (Sensitivity+ Specificity).

Considering only HIV-1 *env* sequences from B subtype specimens, which were the most prevalent in Canada and most represented in our study population (Table 1), the AUC values were slightly increased for two *env* segments, gp120 C2_3 (AUC = 0.810 for subtype B alone compare to 0.805 for all subtypes) and gp120 V3 (AUC = 0.831 for subtype B compare 0.812 for all subtypes), as showed Fig 11 and Table 2.

The performance of sequence-based diversity measures and identifying the most predictable *env* segments were also evaluate for non-B subtypes. As showed in Table 3, the percent complexity, percent diversity, Shannon entropy and number of haplotypes performs better for gp120 C2_1 segment, respectively with an AUC = 0.805, 0.842, 0.844 and 0.826 of each measure. Also, the percent diversity (AUC = 0.821) and number of haplotypes (AUC = 0.849) performed well for *env* gp120 C3_2 segment. Finally, the Percent diversity performed well for gp120 C4 (AUC = 0,819) and gp120 V2 (AUC = 0.84) segments/sub regions. For non-B subtypes, percent diversity seemed to perform well in several *env* segments analyzed (C2_1, C3_2, C4 and V2). But, the lower sequences data, least than 30%, n = 55 (28 recent versus 29 chronic) used in the current study limited the statistical conclusion as well as their performances.

Taking into consideration the Shannon entropy index (*S*) as the best sequence-based diversity measure and *env* gp120-C2_1, gp120-C2-3 and gp120-V3 as the more predictive *env* sub regions/segments, we identified the optimal cut-off values.

Indeed, for all HIV-1 subtypes, the best Shannon entropy index (*S)* cut-off values were as follows: (*S*) = 0.054 for gp120-C2_1, *(S) =* 0.059 for gp120-C2_3 and (*S*) = 0.083 for gp120-V3 (Table 2). Using these cut-off values, the related sensitivity, which determines the true positive rate (recent HIV-1 infected specimens correctly classified), *versus* specificity, which determines the true negative rate (chronic HIV-1 infected specimens correctly classified), was 82% *versus* 69%, 90% *versus* 67% and 87% *versus* 66% for the *env* segments gp120-C2_1, gp120-C2_3 and gp120-V3, respectively.

Moreover, if only sequences from subtype B specimens were considered (Table 2), the sensitivity (recent HIV-1 infected individuals correctly classified) *versus* specificity (chronic HIV-1 infected individuals correctly classified) of the Shannon entropy index (S) was, respectively to 91% *versus* 66%, with a cut-off of (*S)* = 0.059 for *env* gp120 C2_3 and 90% *versus* 66%, respectively, with a cut-off of (*S)* = 0.082 for gp120 V3.

Further analyses excluding p24 positive samples for recency sequences and including only recent infection as determined by MAA and Western Blot positivity, showed that only the Shannon entropy measure of the gp120-V3 segment presented good discriminatory power (AUC = 0.801). This measurement presented 89% of sensitivity to identify recent specimens and 66% of specificity for identifying chronic specimens at a cut-off of (*S)* = 0.0803 (Table 2). On the other hand, when comparing only sequences obtained from p24 positive samples (acute infection), the performance (AUC) and accuracy (sensitivity and specificity) slightly increased for Shannon entropy index (S) measures for three *env* segments (gp120-V3, gp120-C2-1 and gp120-C2-3) (Table 2). Indeed, the Shannon entropy AUC for gp120-V3 was 0.827, which represents a sensitivity of 93% and specificity of 60% at a cut-off value of (*S*) = 0.097 (Table 2). For gp120-C2_1, the AUC was 0.850, which represents a sensitivity of 93% and specificity of 66% at a cut-off of (*S*) = 0.060 (Table 2). Finally, for gp120-C2_3, the AUC was 0.844, representing a sensitivity of 91% and specificity of 71% at a cut-off of (*S*) = 0.048 to correctly identify HIV-1 recency as resumed in Table 2.

## Discussion

In this study, we assessed the performance of 4 sequence-based diversity measures including percent complexity, percent diversity, Shannon entropy and number of haplotypes used either as independent markers or in combinations to predict HIV-1 infection recency. Our analyses focused on 10 subdomains/sub-regions of the HIV-1 envelope gene between gp120-V1 and gp120-C5 and the gp41-ectodomain. These sub-regions or domains are segmented into 13 fragments of 94 to 158 bp.

Because they are too long, the gp120-C2 and gp120-C3 sub-regions are fragmented into 3 and 2 segments, respectively. The choice of these *env* sub-regions was strictly guided by an objective to include all of the gp120 variable regions [70]. Combining multiples segments of them, gp120 C2 (C2_1+C2_2+C2_3) or gp120 C3 (C3_1+C3_2) did not increase discriminatory power of recent HIV-1 infections from chronic ones based on sequences diversities.

We observed that the Shannon entropy measure, which considers the number of reads and proportional representation of each read in individual specimens [42, 43], when calculated for e*nv* gp120-V3, gp120-C2 segment 1 and gp120-C2 segment 3, can correctly distinguish between recently infected and chronically infected individuals with good performance (AUC≥0.8). The fragment lengths of these *env* segments were previously described (i.e., 116 bp for V3, 100 bp for C2_1 and 94 bp for C2_3). These *env* segment sizes suggested that a short fragment of the HIV-1 *env* gene can be useful for predicting HIV-1 recency. Combining Shannon entropy with other measures, such as the percent diversity and/or percent complexity and/or percent complexity within the *env* subdomains, did not markedly increase its predictive value compared to Shannon entropy alone (Figs 9 and 10). This suggests that the single Shannon entropy index as measure performs better than combining with any others diversity measures. It is therefore suggested that the Shannon entropy index (S) within 3 *env* segments (gp120-C2_1, gp120-C2_3 and gp120-V3) as well as HIV-1 subtype B, could be used in public health programs to monitor newly acquired HIV-1 infections in multiple HIV-1 subtype circulating areas.

The utility of viral sequence diversity measurements to determine HIV-1 recency has already been demonstrated [39, 41, 46, 70]. Analysis of segmented regions of the HIV-1 genome to identify the most predictive genomic regions for infection recency has been previously described for *gag* [70]. In this previous study, Wu *et al*., 2015 used a longitudinal subtype C sequence and compared 5 *gag* fragments of 50 bp, 100 bp, 150 bp, 200 bp and 250 bp. They observed that the most predictive regions for recency were those with higher mutation rates, such as gag p17 and p2/p7/p1/p6, compared to more conserved regions, such as *gag* p24 [49]. Furthermore, data used for the latter study were derived from first generation sequencing, which probably underestimates viral diversity since minor variants need to represent more than 20% of the total population to be detected using this technique [45]. The NGS approach used in our current study has been shown to be more sensitive and may offer the possibility of detecting minor HIV-1 variants/quasi-species that are present in less than 1% of the viral population sampled[42, 43]. We have decided to screen the HIV-1 envelope sequence diversity (the gp120 and gp41 regions) as this gene include the most variable regions of the HIV genome.

It is therefore more representative of viral diversification over time as they undergo constant selective pressure from the immune system [71, 72].

Here, when using the median calculation and the frequency distribution curves, we showed that recently infected individuals presented significantly less *env* sequence-based diversity than chronically infected ones. Our data confirmed previous observations indicating that sequences from recently infected populations are more homogeneous than those from chronically infected populations[50]. These differences were clearly observed by the Shannon entropy, percent diversity and number of haplotypes measures, while the percent complexity was not clearly different between the two groups for 3 *env* segments (S2, S3 and S4 Tables). These results confirm previous results by Cousins *et al*., 2012 [42], who analyzed mostly subtype D *env* gp41 fragments, while our specimen collection contained predominantly subtype B and evaluated 13 shorts segments of HIV-1 *env* gp120 and gp41 (from 94bp to 158bp). The analysis of shorts segments of the *env* gene is interesting from a technical standpoint since amplification and library preparation from shorts DNA fragments during sequencing is time-saving (i.e., no fragmentation step required) and can be achieved at a lower cost on Illumina MiSeq platforms.

The performance of the sequence-based diversity measure using the AUC of ROC analysis in our current study globally supports previous investigations by Moyo *et al*., 2016 [73], However this study were conducted on the *gag* and *env* regions from SGA of HIV-1 subtype C using the pairwise genetic distance (PwD) or percent diversity as measure of *env* gp120-V1C5 fragment. The authors determinates an AUC of 0.83 at 130 days of infection[73], which is considered to be good discriminatory power.

Comparatively to Moyo *et al*., 2016 approaches, the current study determines moderate discriminatory power of *env* gp120-V1C5 with Shannon entropy index of (AUC = 0.765 [0.747–0,784] and for Percent diversity (AUC = 0,704 [0,684–0,723]. Comparatively to our results, sequences data used (HIV-1 C subtype compare to predominant B subtype) may probably impact performance of sequences based diversity estimating. In the current study, HIV-1 C subtype represented less than 4% (n = 9) of study population so that, we cannot make performant statistical analyses and address objective comparison with Moyo *et al*., 2016 study. However, our finding may contribute to knowledges with identification of very shorts predictable *env* segments of B and non-B subtypes populations. Also, we have identified a best sequenced-based diversity measure (Shannon entropy) which performed well for accurate identifying of HIV-1 recency. However, it would be subjective to address a comparison using the same algorithm with similar segmented regions if we must consider the HIV-1 subtype on these published data and compare accuracy of both approaches. Futures studies using the same sequences data would be necessary.

In our current study, we found that only the Shannon entropy index presented good discriminatory power for three *env* segments (gp120-C2-1, gp120-C2_3 and gp120-V3), while the percent diversity measure presented fair or poor discrimination for a few *env* segments. These differences could be linked to the fact that, in our study, shorts segments of the *env* gene were analyzed and that our specimen collection was mostly composed of HIV-1 subtype B. Nevertheless, by 130 days of infection, the sensitivity (true recent infected individuals correctly classified) of Moyo *et al*., 2016 [73] study was 79.37% and established (specificity) 72.57% at PwD cut-off of 0.005. This sensitivity (79.37%) was less than that found in our study for Shannon entropy, which provided a sensitivity of 87% and specificity of 66% in gp120 V3, 82% *versus* 69% in gp120 C2_1 and 90% *versus* 67% in gp120 C2_3.

These differences indicate that the Shannon entropy index performs better for the identification of HIV-1 recency regarding the highest proportion of recently HIV-1 infected individuals correctly identified comparatively to the chronically infected ones.

Yang *et al*.,2012 also used the PwD to identify recently HIV-1 subtype B and CRF07_BC infected individuals using the *env* gp120-C2V5 region. In this previous study, authors found an AUC = 0.97 at a sensitivity of 90 to 95% *versus* specificity of 78.8% in population of (n = 160 for recent *versus* 264 chronic infected individuals) at a PwD cut-off value of 0.24 by 150–350 days of infection [74]. We observed the similar performance with true recent HIV-1 infected individuals correctly classified (sensitivity) *versus* chronic ones (specificity) of 90% *versus* 66% in gp120 V3 and 91% *versus* 66% in gp120_C2_3 for the Shannon entropy measure.

The HIV-1 recency power increased for acutely infected individuals′ samples (p24+ WB-) compared to chronically infected individuals’ samples over that of recently infected individuals < 136 days (WB+ and MAA determination) [24] compared to chronically infected individuals, as shown in Table 2. This is consistent with *env* gene diversification (Fiebig stage) following HIV-1 transmission, as shown by Keele *et al*., 2008 [50], and confirms the greater HIV-1 *env* sequence homogeneity and diversity increasing in the acute to recent stage of infection and the highest *env* sequence diversity (heterogeneity) in chronic/late stage of infection.

In summary, our current study shows that the Shannon entropy of HIV-1 *env* gp120-V3 and gp120 C2 segments 1 and 3 correctly predicts recent HIV-1 infections with performant accuracy. Importantly, HIV-1 *env* gp120-V3 was shown to be the best predictor of HIV-1 recency for the B and non-B subtypes and percent diversity for non-B alones. Sequencing of the V3 loop is often performed to determine HIV-1 co-receptor tropism [75] allowing combination with this method to obtain recency data.

Therefore, we suggest that targeted sequencing of short *env* segments can be useful for determining HIV-1 recency with more sensitivity than sequencing the entire *env* gene and may represent an option that minimizes both cost and time factors compared to full-length HIV-1 envelope amplification and sequencing, which constitute a serious limitation for the use of sequence-based diversity for HIV-1 recency identification.

## Supporting information

S1 Dataset Viral sequences data qualifiers. (XLSX)

S1 Table Sequence based diversity measures calculation methods. (PDF)

S2 Table Sequence-based diversity measures expressed as the median/IQR and calculated from NGS of 7 segments representing 4 selected HIV-1 *env* GP120 conserved subdomains. (PDF)

S3 Table Sequence-based diversity measures expressed as the median/IQR and calculated from NGS of 5 segments representing 5 selected HIV-1 *env* GP120 variable loops and 1 segment for a part of the gp41ectodomain. (PDF)

S4 Table Sequence-based diversity measures expressed as the mean with student t-test results comparing recent versus chronic HIV infected sequences by 13 *env* segments. (PDF)
