# Supplementary material for: HIV-1 envelope sequence-based diversity measures for identifying recent infections
Source: PLoS One. 2017 Dec 28;12(12):e0189999. doi: 10.1371/journal.pone.0189999 (PMC5746209; doi:10.1371/journal.pone.0189999)
Supplement: S1 Table — (PDF) [file pone.0189999.s002.pdf]

S1 Table: Method of Calculation of sequence-based diversity measures

TABLE 1 Description of sequence-based diversity measures

Diversity measure	Method of calculation
% diversity	Average pairwise genetic distance was calculated using the maximum composite likelihood model. MEGA 7.0.14 (www.megasoftware.net) was used to analyze gap-stripped sequence alignments. This value is reported as a percentage.
% complexity	[(no. of distinct variants)/(total no. of reads)] × 100
Shannon entropy (S)	$S = -\left(\frac{1}{\log N}\right)\sum_{i=1}^{n} p_i \log p_i$ where p_i is the proportion of reads consisting of each unique sequence pattern, N is the total no. of sequences, and n is the no. of distinct sequences

Sequence-based measures were calculated using the method previously described by Cousins MM, Ou SS, Wawer MJ, Munshaw S, Swan D, Magaret CA, Mullis CE, Serwadda D, Porcella SF, Gray RH, Quinn TC, Donnell D, Eshleman SH, Redd AD. Comparison of a high-resolution melting assay to next-generation sequencing for analysis of HIV diversity. J Clin Microbiol. 2012 Sep;50(9):3054-9. doi:10.1128/JCM.01460-12.
