# Supplementary material for: HIV-1 envelope sequence-based diversity measures for identifying recent infections
Source: PLoS One. 2017 Dec 28;12(12):e0189999. doi: 10.1371/journal.pone.0189999 (PMC5746209; doi:10.1371/journal.pone.0189999)
Supplement: S2 Table — (PDF) [file pone.0189999.s003.pdf]

S2 Table. Sequence-based diversity measures expressed as the median/IQR and calculated from NGS of 7 segments representing 4 selected HIV-1 *env* GP120 conserved subdomains.

**HIV-1 *env* segment**	**Sequence-based diversity measure**	**Recent HIV-1 infected population**			**Chronic HIV-1 infected population**		
		Median	IQR	Nb of sample analyzed (n)	Median	IQR	Nb of sample analyzed (n)
GP120-C2_1	Percent diversity	0.01	[0.00-0.09]	134	0.01	[0.00-0.18]	115
	Percent complexity	0.00	[0.00-0.00]	134	0.00	[0.00-0.02]	115
	Shannon Entropy	0.01	[0.00-0.16	134	0.10	[0.00-0.26]	115
	Number of Haplotypes	3	[1-13]	134	5	[1-20]	115
GP120-C2_2	Percent diversity	0.01	[0.00-0.06]	134	0.01	[0.00-0.08]	115
	Percent complexity	0.00	[0.00-0.00]	134	0.00	[0.00-0.00]	115
	Shannon Entropy	0.01	[0.00-0.13]	134	0.07	[0.00-0.25]	115
	Number of Haplotypes	3	[1-10]	134	5	[1-17]	115
GP120-C2_3	Percent diversity	0.01	[0.00-0.14]	134	0.02	[0.00-0.14]	115
	Percent complexity	0.00	[0.00-0.00]	134	0.00	[0.00-0.00]	115
	Shannon Entropy	0.01	[0.00-0.18]	134	0.09	[0.00-0.32]	115
	Number of Haplotypes	3	[1-12]	134	6	[1-25]	115
GP120-C3_1	Percent diversity	0.01	[0.00-0.16]	134	0.02	[0.00-0.16]	115
	Percent complexity	0.00	[0.00-0.00]	134	0.00	[0.00-0.00]	115
	Shannon Entropy	0.01	[0.00-0.19]	134	0.09	[0.00-0.30]	115
	Number of Haplotypes	3	[1-14]	134	5	[1-28]	115
GP120-C3_2	Percent diversity	0.01	[0.00-0.16]	134	0.02	[0.00-0.16]	115
	Percent complexity	0.00	[0.00-0.00]	134	0.00	[0.00-0.01]	115
	Shannon Entropy	0.02	[0.00-0.35]	134	0.12	[0.00-0.25]	115
	Number of Haplotypes	3	[1-5]	134	8	[1-12]	115
GP120-C4	Percent diversity	0.01	[0.00-0.09]	134	0.02	[0.00-0.19]	115
	Percent complexity	0.00	[0.00-0.00]	134	0.00	[0.00-0.00]	115
	Shannon Entropy	0.01	[0.00-0.25]	134	0.09	[0.00-0.27]	115
	Number of Haplotypes	3	[1-19]	134	6	[1-22]	115
GP120-C5	Percent diversity	0.01	[0.00-0.11]	134	0.01	[0.00-0.10]	115
	Percent complexity	0.00	[0.00-0.01]	134	0.00	[0.00-0.00]	115
	Shannon Entropy	0.02	[0.00-0.26]	134	0.11	[0.00-0.34]	115
	Number of Haplotypes	4	[1-18]	134	6	[1-22]	115

HIV-1 *env* segment	Sequence-based diversity measure	Recent HIV-1 infected population			Chronic HIV-1 infected population		
		Median	IQR	Nb of sample analyzed (n)	Median	IQR	Nb of sample analyzed (n)
GP120-C2_1	Percent diversity	0.01	[0.00-0.09]	134	0.01	[0.00-0.18]	115
	Percent complexity	0.00	[0.00-0.00]	134	0.00	[0.00-0.02]	115
	Shannon Entropy	0.01	[0.00-0.16	134	0.10	[0.00-0.26]	115
	Number of Haplotypes	3	[1-13]	134	5	[1-20]	115
GP120-C2_2	Percent diversity	0.01	[0.00-0.06]	134	0.01	[0.00-0.08]	115
	Percent complexity	0.00	[0.00-0.00]	134	0.00	[0.00-0.00]	115
	Shannon Entropy	0.01	[0.00-0.13]	134	0.07	[0.00-0.25]	115
	Number of Haplotypes	3	[1-10]	134	5	[1-17]	115
GP120-C2_3	Percent diversity	0.01	[0.00-0.14]	134	0.02	[0.00-0.14]	115
	Percent complexity	0.00	[0.00-0.00]	134	0.00	[0.00-0.00]	115
	Shannon Entropy	0.01	[0.00-0.18]	134	0.09	[0.00-0.32]	115
	Number of Haplotypes	3	[1-12]	134	6	[1-25]	115
GP120-C3_1	Percent diversity	0.01	[0.00-0.16]	134	0.02	[0.00-0.16]	115
	Percent complexity	0.00	[0.00-0.00]	134	0.00	[0.00-0.00]	115
	Shannon Entropy	0.01	[0.00-0.19]	134	0.09	[0.00-0.30]	115
	Number of Haplotypes	3	[1-14]	134	5	[1-28]	115
GP120-C3_2	Percent diversity	0.01	[0.00-0.16]	134	0.02	[0.00-0.16]	115
	Percent complexity	0.00	[0.00-0.00]	134	0.00	[0.00-0.01]	115
	Shannon Entropy	0.02	[0.00-0.35]	134	0.12	[0.00-0.25]	115
	Number of Haplotypes	3	[1-5]	134	8	[1-12]	115
GP120-C4	Percent diversity	0.01	[0.00-0.09]	134	0.02	[0.00-0.19]	115
	Percent complexity	0.00	[0.00-0.00]	134	0.00	[0.00-0.00]	115
	Shannon Entropy	0.01	[0.00-0.25]	134	0.09	[0.00-0.27]	115
	Number of Haplotypes	3	[1-19]	134	6	[1-22]	115
GP120-C5	Percent diversity	0.01	[0.00-0.11]	134	0.01	[0.00-0.10]	115
	Percent complexity	0.00	[0.00-0.01]	134	0.00	[0.00-0.00]	115
	Shannon Entropy	0.02	[0.00-0.26]	134	0.11	[0.00-0.34]	115
	Number of Haplotypes	4	[1-18]	134	6	[1-22]	115

Median differences between recent and chronic HIV-1 infected individuals for 4 sequence-based diversity measures of 7 env segments.

IQR: interquartile range.
