# Supplementary material for: HIV-1 envelope sequence-based diversity measures for identifying recent infections
Source: PLoS One. 2017 Dec 28;12(12):e0189999. doi: 10.1371/journal.pone.0189999 (PMC5746209; doi:10.1371/journal.pone.0189999)
Supplement: S3 Table — (PDF) [file pone.0189999.s004.pdf]

S3 Table. Sequence-based diversity measures expressed as the median/ IQR and calculated from NGS of 5 segments representing 5 selected HIV-1 *env* GP120 loop and 1 segment for a part of the GP41ectodomain (NHR).

**HIV-1 *env* segment**	**Sequence-based diversity measure**	**Recent HIV-1 infected population**			**Chronic HIV-1 infected population**		
		Median	IQR	Number of sample analyzed (n)	Median	IQR	Number of sample analyzed (n)
GP120-V1	Percent diversity	0.01	[0.00-0.05]	63	0.02	[0.00-0.09]	35
	Percent complexity	0.00	[0.00-0.18]	63	0.01	[0.00-0.25]	35
	Shannon Entropy	0.05	[0.00-0.31]	63	0.11	[0.00-0.54]	35
	Number of Haplotypes	4	[1-7]	63	6	[1-12]	35
GP120-V2	Percent diversity	0.01	[0.00-0.06]	102	0.01	[0.01-0.09]	77
	Percent complexity	0.00	[0.00-0.01]	102	0.00	0.00-0.02	77
	Shannon Entropy	0.02	[0.00-0.26]	102	0.12	[0.00-0.32]	77
	Number of Haplotypes	4	[1-13]	102	8	[2-26]	77
GP120-V3	Percent diversity	0.01	[0.00-0.16]	134	0.02	[0.00-0.17]	115
	Percent complexity	0.00	[0.00-0.00]	134	0.00	[0.00-0.00]	115
	Shannon Entropy	0.02	[0.00-0.24]	134	0.12	[0.00-0.32]	115
	Number of Haplotypes	3	[1-16]	134	7	[2-22]	115
GP120-V4	Percent diversity	0.01	[0.00-0.13]	134	0.02	[0.00-0.14]	115
	Percent complexity	0.00	[0.00-0.00]	134	0.00	[0.00-0.00]	115
	Shannon Entropy	0.01	[0.00-0.23]	134	0.10	[0.00-0.27]	115
	Number of Haplotypes	3	[1-17]	134	6	[2-23]	115
GP120-V5	Percent diversity	0.02	[0.00-0.14]	134	0.03	[0.00-0.17]	115
	Percent complexity	0.00	[0.00-0.00]	134	0.00	[0.00-0.00]	115
	Shannon Entropy	0.01	[0.00-0.14]	134	0.06	[0.00-0.25]	115
	Number of Haplotypes	3	[1-13]	134	4	[1-21]	115
GP41-NHR	Percent diversity	0.01	[0.00-0.05]	131	0.01	[0.01-0.05]	107
	Percent complexity	0.00	[0.00-0.08]	131	0.00	[0.00-0.13]	107
	Shannon Entropy	0.05	[0.00-0.30]	131	0.13	[0.00-0.42]	107
	Number of Haplotypes	5	[1-20]	131	7	[2-35]	107

Median differences between recent and chronic infected individuals 4 sequence-based diversity measures of 6 env segments.
IQR: interquartile range.
