# Supplementary material for: HIV-1 envelope sequence-based diversity measures for identifying recent infections
Source: PLoS One. 2017 Dec 28;12(12):e0189999. doi: 10.1371/journal.pone.0189999 (PMC5746209; doi:10.1371/journal.pone.0189999)
Supplement: S4 Table — (PDF) [file pone.0189999.s005.pdf]

S4 Table: Sequence-based diversity measures expressed as the mean with student t-test results comparing recent versus chronic HIV infected sequences by 13 env segments.

Env segment	Diversity measure	Infection	N	Mean	Std. Deviation	Std. Error Mean	t-test	P value	Observations
**GP120 C2_1**	**Percent complexity**	**RECENT**	**134**	**4,31E-04**	**6,15E-04**	**5,31E-05**	**-2,79**	**0,006**	**NS**
		CHRONIC	115	1,03E-03	2,38E-03	2,22E-04			
	Percent diversity	RECENT	134	1,27E-02	1,15E-02	9,92E-04	-5,44	0,000	S
		CHRONIC	115	2,41E-02	2,08E-02	1,94E-03			
	Shannon entropy index	RECENT	134	3,03E-02	3,51E-02	3,03E-03	-10,1	0,000	S
		CHRONIC	115	1,05E-01	7,62E-02	7,11E-03			
	Number of haplotypes	RECENT	134	3,25E+00	2,00E+00	1,73E-01	-8,23	0,000	S
		CHRONIC	115	6,96E+00	4,74E+00	4,42E-01			
**GP120 C2_2**	Percent complexity	RECENT	134	1,76E-04	2,08E-04	1,80E-05	-4,04	0,000	S
		CHRONIC	115	3,59E-04	4,73E-04	4,41E-05			
	Percent diversity	RECENT	134	1,21E-02	8,47E-03	7,32E-04	-6,27	0,000	S
		CHRONIC	115	2,13E-02	1,42E-02	1,32E-03			
	Shannon entropy index	RECENT	134	2,54E-02	3,08E-02	2,66E-03	-8,83	0,000	S
		CHRONIC	115	8,21E-02	6,64E-02	6,20E-03			
	Number of haplotypes	RECENT	134	3,09E+00	1,88E+00	1,62E-01	-7,5	0,000	S
		CHRONIC	115	5,97E+00	3,95E+00	3,68E-01			
**GP120 C2_3**	Percent complexity	RECENT	134	2,55E-04	3,38E-04	2,92E-05	-4,35	0,000	S
		CHRONIC	115	5,84E-04	7,95E-04	7,41E-05			
	Percent diversity	RECENT	134	1,44E-02	1,75E-02	1,51E-03	-4,94	0,000	S
		CHRONIC	115	2,86E-02	2,73E-02	2,55E-03			
	Shannon entropy index	RECENT	134	2,80E-02	3,48E-02	3,00E-03	-10,1	0,000	S
		CHRONIC	115	1,00E-01	7,39E-02	6,89E-03			
	Number of haplotypes	RECENT	134	3,23E+00	1,98E+00	1,71E-01	-7,84	0,000	S
		CHRONIC	115	6,79E+00	4,80E+00	4,48E-01			

**GP120 C3_1**	Percent complexity	RECENT	134	2,56E-04	4,70E-04	4,06E-05	-2,28	0,023	S
		CHRONIC	115	3,89E-04	4,48E-04	4,17E-05			
	Percent diversity	RECENT	134	2,03E-02	1,91E-02	1,65E-03	-5,17	0,000	S
		CHRONIC	115	3,69E-02	3,10E-02	2,89E-03			
	Shannon entropy index	RECENT	134	3,01E-02	3,45E-02	2,98E-03	-9,16	0,000	S
		CHRONIC	115	9,29E-02	7,00E-02	6,53E-03			
	Number of haplotypes	RECENT	134	3,36E+00	1,99E+00	1,72E-01	-6,96	0,000	S
		CHRONIC	115	6,52E+00	4,81E+00	4,48E-01			
**GP120 C3_2**	**Percent complexity**	**RECENT**	**134**	**5,49E-04**	**1,19E-03**	**1,03E-04**	**-1,93**	**0,054**	**NS**
		CHRONIC	115	8,40E-04	1,19E-03	1,11E-04			
	Percent diversity	RECENT	134	1,74E-02	2,18E-02	1,88E-03	-6,08	0,000	S
		CHRONIC	115	3,72E-02	2,96E-02	2,76E-03			
	Shannon entropy index	RECENT	134	4,46E-02	5,68E-02	4,90E-03	-8,49	0,000	S
		CHRONIC	115	1,15E-01	7,34E-02	6,84E-03			
	Number of haplotypes	RECENT	134	4,01E+00	3,38E+00	2,92E-01	-7,88	0,000	S
		CHRONIC	115	8,18E+00	4,92E+00	4,59E-01			
**GP120 C4**	Percent complexity	RECENT	134	3,64E-04	6,45E-04	5,57E-05	-2,53	0,012	S
		CHRONIC	115	5,60E-04	5,67E-04	5,29E-05			
	Percent diversity	RECENT	134	2,01E-02	1,69E-02	1,46E-03	-4,75	0,000	S
		CHRONIC	115	3,48E-02	3,09E-02	2,88E-03			
	Shannon entropy index	RECENT	134	3,62E-02	4,51E-02	3,90E-03	-8,85	0,000	S
		CHRONIC	115	1,05E-01	7,53E-02	7,02E-03			
	Number of haplotypes	RECENT	134	3,92E+00	2,76E+00	2,38E-01	-6,83	0,000	S
		CHRONIC	115	7,35E+00	5,00E+00	4,66E-01	-6,56		
**GP120 C5**	Percent complexity	RECENT	134	8,42E-04	1,43E-03	1,23E-04	-2,54	0,012	S
		CHRONIC	115	1,28E-03	1,26E-03	1,18E-04			
	Percent diversity	RECENT	134	1,33E-02	1,21E-02	1,05E-03	-3,06	0,002	S
		CHRONIC	115	1,85E-02	1,46E-02	1,36E-03			
	Shannon entropy index	RECENT	134	4,47E-02	4,88E-02	4,22E-03	-7,95	0,000	S
		CHRONIC	115	1,14E-01	8,60E-02	8,02E-03			

	Number of haplotypes	RECENT	134	4,28E+00	2,55E+00	2,21E-01	-6,05	0,000	S
		CHRONIC	115	7,28E+00	5,03E+00	4,69E-01			
**GP41 ECD (NHR)**	Percent complexity	RECENT	131	6,80E-03	9,95E-03	8,69E-04	-4,1	0,000	S
		CHRONIC	107	1,54E-02	2,15E-02	2,07E-03			
	Percent diversity	RECENT	131	1,06E-02	5,94E-03	5,19E-04	-3,47	0,001	S
		CHRONIC	107	1,33E-02	6,02E-03	5,82E-04			
	Shannon entropy index	RECENT	131	7,04E-02	5,70E-02	4,98E-03	-7,41	0,000	S
		CHRONIC	107	1,54E-01	1,12E-01	1,08E-02			
	Number of haplotypes	RECENT	131	5,53E+00	2,86E+00	2,50E-01	-5,56	0,000	S
		CHRONIC	107	8,70E+00	5,72E+00	5,53E-01			
**GP120 V1**	**Percent complexity**	**RECENT**	**63**	**1,29E-02**	**2,61E-02**	**3,29E-03**	**-1,84**	**0,069**	**NS**
		CHRONIC	35	2,65E-02	4,73E-02	7,99E-03			
	Percent diversity	RECENT	63	1,98E-02	7,04E-03	8,87E-04	-4,36	0,000	S
		CHRONIC	35	3,06E-02	1,72E-02	2,90E-03			
	Shannon entropy index	RECENT	63	7,01E-02	6,11E-02	7,70E-03	-3,48	0,001	S
		CHRONIC	35	1,35E-01	1,24E-01	2,10E-02			
	Number of haplotypes	RECENT	63	5,76E+00	5,08E+00	6,40E-01	-2,76	0,007	S
		CHRONIC	35	9,57E+00	8,63E+00	1,46E+00			
**GP120 V2**	Percent complexity	RECENT	102	1,25E-03	2,08E-03	2,06E-04	-3,87	0,000	S
		CHRONIC	77	3,31E-03	4,80E-03	5,47E-04			
	Percent diversity	RECENT	102	1,16E-02	9,62E-03	9,52E-04	-6,47	0,000	S
		CHRONIC	77	2,64E-02	2,04E-02	2,32E-03			
	Shannon entropy index	RECENT	102	4,85E-02	5,12E-02	5,07E-03	-7,59	0,000	S
		CHRONIC	77	1,29E-01	9,03E-02	1,03E-02			
	Number of haplotypes	RECENT	102	4,12E+00	2,34E+00	2,32E-01	-7,22	0,000	S
		CHRONIC	77	8,27E+00	5,15E+00	5,87E-01			

**GP120 V3**	Percent complexity	RECENT	134	5,13E-04	8,34E-04	7,21E-05	-4,2	0,000	S
		CHRONIC	115	1,06E-03	1,20E-03	1,12E-04			
	Percent diversity	RECENT	134	1,57E-02	1,94E-02	1,67E-03	-5,87	0,000	S
		CHRONIC	115	3,48E-02	3,13E-02	2,92E-03			
	Shannon entropy index	RECENT	134	3,92E-02	4,49E-02	3,88E-03	-10,2	0,000	S
		CHRONIC	115	1,24E-01	8,29E-02	7,73E-03			
	Number of haplotypes	RECENT	134	3,90E+00	2,65E+00	2,29E-01	-8,97	0,000	S
		CHRONIC	115	8,26E+00	4,85E+00	4,53E-01			
**GP120 V4**	Percent complexity	RECENT	134	4,03E-04	6,82E-04	5,89E-05	-2,69	0,008	S
		CHRONIC	115	6,42E-04	7,20E-04	6,71E-05			
	Percent diversity	RECENT	134	1,58E-02	1,63E-02	1,41E-03	-5,56	0,000	S
		CHRONIC	115	3,17E-02	2,79E-02	2,60E-03			
	Shannon entropy index	RECENT	134	3,60E-02	4,67E-02	4,03E-03	-8,86	0,000	S
		CHRONIC	115	1,05E-01	7,52E-02	7,01E-03			
	Number of haplotypes	RECENT	134	3,86E+00	2,73E+00	2,35E-01	-6,78	0,000	S
		CHRONIC	115	7,17E+00	4,82E+00	4,50E-01			
**GP120 V5**	**Percent complexity**	**RECENT**	**134**	**1,76E-04**	**4,19E-04**	**3,62E-05**	**-1,92**	**0,057**	**NS**
		CHRONIC	115	2,67E-04	3,15E-04	2,94E-05			
	Percent diversity	RECENT	134	2,78E-02	2,08E-02	1,79E-03	-4,76	0,000	S
		CHRONIC	115	4,61E-02	3,83E-02	3,58E-03			
	Shannon entropy index	RECENT	134	2,39E-02	3,00E-02	2,59E-03	-8,16	0,000	S
		CHRONIC	115	7,48E-02	6,46E-02	6,02E-03			
	Number of haplotypes	RECENT	134	3,22E+00	2,14E+00	1,85E-01	-5,1	0,000	S
		CHRONIC	115	5,15E+00	3,71E+00	3,46E-01			

Sequence based diversity means of recent compare to chronic HIV-1 infected individuals with t-test results.

NS: *Statistically nonsignificant* ***S:*** *Statistically significant*
